# CT findings of adnexal torsion: A matched case-control study

**DOI:** 10.1371/journal.pone.0200190

**Published:** 2018-07-11

**Authors:** Myoung Seok Lee, Min Hoan Moon, Hyunsik Woo, Chang Kyu Sung, Sohee Oh, Hye Won Jeon, Taek Sang Lee

**Affiliations:** 1 Department of Radiology, SMG-SNU Boramae Medical Center, Seoul National University College of Medicine, Seoul, Korea; 2 Department of Biostatistics, SMG-SNU Boramae Medical Center, Seoul National University College of Medicine, Seoul, Korea; 3 Department of Obstetrics and Gynecology, SMG-SNU Boramae Medical Center, Seoul National University College of Medicine, Seoul, Korea; University of Insubria, ITALY

## Abstract

**Objective:**

The purpose of our study was to assess computed tomographic (CT) findings of adnexal torsion through a matched case-control analysis.

**Materials and methods:**

This retrospective, single-institution case-control study included 43 women with adnexal torsion and 43 age- and ovarian mass-matched control women. CT images were evaluated independently by two readers for the following: prominent peripheral follicles, uterine deviation, thickened pedicles, a whirl sign, and a navel sign. Comparisons of CT findings were performed using the Chi square test and receiver operating characteristic (ROC) curves were obtained to assess the diagnostic performance. Differences between the areas under the ROC curves (AUCs) were compared by using a Delong test.

**Results:**

The CT findings significant for adnexal torsion were uterine deviation toward the side of the affected ovary (*P* = < .01 for reader 1 and *P* = .02 for reader 2) and thickened pedicles with ancillary findings including a whirl sign, a navel sign, and uterine deviation facing thickened pedicles (*P* < .01 for both readers). Thickened pedicles with ancillary findings had the highest diagnostic accuracy, as measured with ROC curves (AUC, 0.86 in reader 1 and 0.85 in reader 2). Combining uterine deviation toward the side of the affected ovary with thickened pedicles with ancillary findings did not increase the performance relative to that of thickened pedicles with ancillary findings alone.

**Conclusions:**

Thickened pedicles with ancillary findings including a whirl sign, a navel sign, and uterine deviation facing thickened pedicles could be helpful for the diagnosis of adnexal torsion.

## Introduction

Adnexal torsion is a gynecologic emergency accounting for 2.7% of female acute pelvic pain [1]. Transvaginal ultrasound (US) has been considered to be the diagnostic modality of choice to assess torsion [2, 3] but computed tomography (CT) may often be the initial imaging modality because the presentation of adnexal torsion is nonspecific and overlapping with other more commonly encountered acute abdominal conditions such as appendicitis or ureteral stones [4–6]. As prompt diagnosis of adnexal torsion may reduce the risk of irreversible ovarian ischemia, it is important to be familiar with the CT findings of adnexal torsion for a timely diagnosis and to improve clinical outcomes. Several previous studies have reported CT findings of adnexal torsion [4, 5, 7, 8]. However, most of the previous studies were descriptive studies performed in a selected series of cases, carrying the risk of biased results and limited generalizability due to the lack of controls. Furthermore, previous studies did not consider the effect of an ovarian mass in the assessment of CT findings of adnexal torsion. Most cases of adnexal torsion are associated with ovarian masses [9, 10]. However, given that most ovaries containing a mass are not torsed, an ovarian mass cannot be a specific finding of adnexal torsion but it can affect CT findings [11]. For this reason, we made additional efforts to eliminate the confounding caused by ovarian masses in this retrospective case-control study. Therefore, our objective was to move beyond an ovarian mass as a CT predictor of adnexal torsion and to identify additional CT findings that could be helpful in the diagnosis of adnexal torsion.

## Materials and methods

This retrospective, single-institution, case–control study was approved by our institutional review board of Seoul Metropolitan Government Seoul National University Boramae Medical Center (16-2017-26), and informed consent was waived due to the retrospective nature of the study.

### Study population

**Fig 1:**
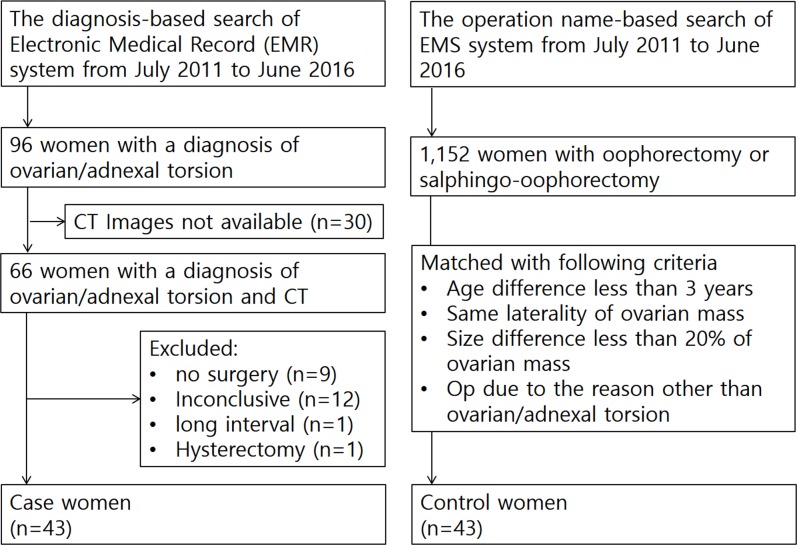
Flowchart of the study population selection for this retrospective mass-matched case-control study.

The diagnosis-based search engine of our electronic medical record (EMR) system revealed 96 consecutive women with a diagnosis of ovarian/adnexal torsion from July 2011 to June 2016. Among them, 66 women who underwent multiphase CT scans including both unenhanced phase and portal venous phase were included in the study. On the basis of retrospective medical record review, 23 women were excluded from the 66 women for the following reasons: no surgical exploration (n = 9), inconclusive for adnexal torsion on surgical records (n = 12), long interval between CT imaging and surgery (n = 1), and previous hysterectomy (n = 1). Finally, 43 women (mean age, 40.8 years; range, 13–84 years) compose the case women who had met study criteria of receiving multiphase CT scans, surgically confirmed diagnosis of ovarian/adnexal torsion, and no hysterectomy. A total of 43 control women (mean age, 41.3 years; range, 16–87 years) were selected from 1,152 consecutive women who underwent oophorectomy or salphingo-oophrectomy during the same study period. The selection of control women was performed as follows: 1) 1,152 women were arranged in the order of age. 2) One author (H.W.) selected a candidate in order, who matched with a case woman for age (± 3 years), laterality of ovarian mass, and size of ovarian mass. The size of the ovarian mass was assessed on axial CT scans and less than 20% difference based on the smaller one was considered matched. 3) Selection as a control was finalized after confirming that the operation was performed due to a reason other than ovarian/adnexal torsion (Fig 1). The median delay between CT imaging and surgery was 1 day (range, 0–13 days) in case women and 10 days (range, 0–76 days) in control women.

Clinical and pathologic data for the study population were extracted from our EMR system, with additional retrospective medical record review. The following clinical findings related to the abdominal pain were recorded: (a) presence of abdominal pain, (b) duration of abdominal pain, and (c) intensity of abdominal pain. At our institution, pain intensity was quantified and recorded by using an 11-point numerical rating scale in which 0 means no pain and 10 means the worst pain possible [12, 13].

### CT acquisition technique

CT images were obtained using three different types of scanners: (a) LightSpeed Pro 16 (GE Medical Systems, Milwaukee, Wi, USA) with a detector configuration, 16 * 1.25 mm; tube voltage, 120 kVp; noise index, 12.35 with automatic exposure control (smart mA, GE Healthcare, Milwaukee, WI, USA); gantry rotation period, 0.6 second; pitch factor, 1.375; table speed, 27.5 mm per rotation, reconstructed section width, 3.75 mm; and reconstructed section interval, 3.75 mm [n = 55], (b) Brilliance 64 (Philips, Cleveland, OH, USA) with detector configuration, 64 * 0.625 mm; tube voltage, 120 kVp; 300 reference mAs with automatic exposure control (DoseRight ACS, Philips, Cleveland, Ohio); gantry rotation period, 0.75 second; pitch factor, 1; table speed, 40 mm per rotation, reconstructed section width, 3 mm; and reconstructed section interval, 3 mm [n = 19], and (c) Ingenuity 128 (Philips, Cleveland, OH, USA) with detector configuration, 64 * 0.625 mm; tube voltage, 120 kVp; dose index, 24 with automatic exposure control (DoseRight ACS, Philips, Cleveland, OH, USA); gantry rotation period/pitch factor, automatic scan time; reconstructed section width, 3 mm; and reconstructed section interval, 3 mm [n = 12]. No oral contrast material was given for gastrointestinal tract opacification. Nonionic low-osmolar iodine contrast material containing 350 mg of iodine per milliliter (iohexol, Omnihexol 350; Korea United Pharm Co, Seoul, Korea) was intravenously administered in a volume of 1.5 mL/kg by using a power injector (Optivantage DH; Mallinckrodt Imaging Solutions, Hazelwood, MO, USA) at a rate of 2–3 mL/sec. Women underwent multi-phase CT scans including at least two phases (unenhanced and portal venous phase). The scanning delay for the portal venous phase was 90 seconds after initiation of contrast material injection and the phases were obtained craniocaudally from the diaphragmatic dome to the inferior margin of the symphysis pubis. Coronal images for the portal venous phase were reconstructed in 30 of 43 case women (69.8%) and 35 of 43 control women (81.4%), with a section width/interval of 3 mm / 3 mm.

### Image analysis

**Fig 2:**
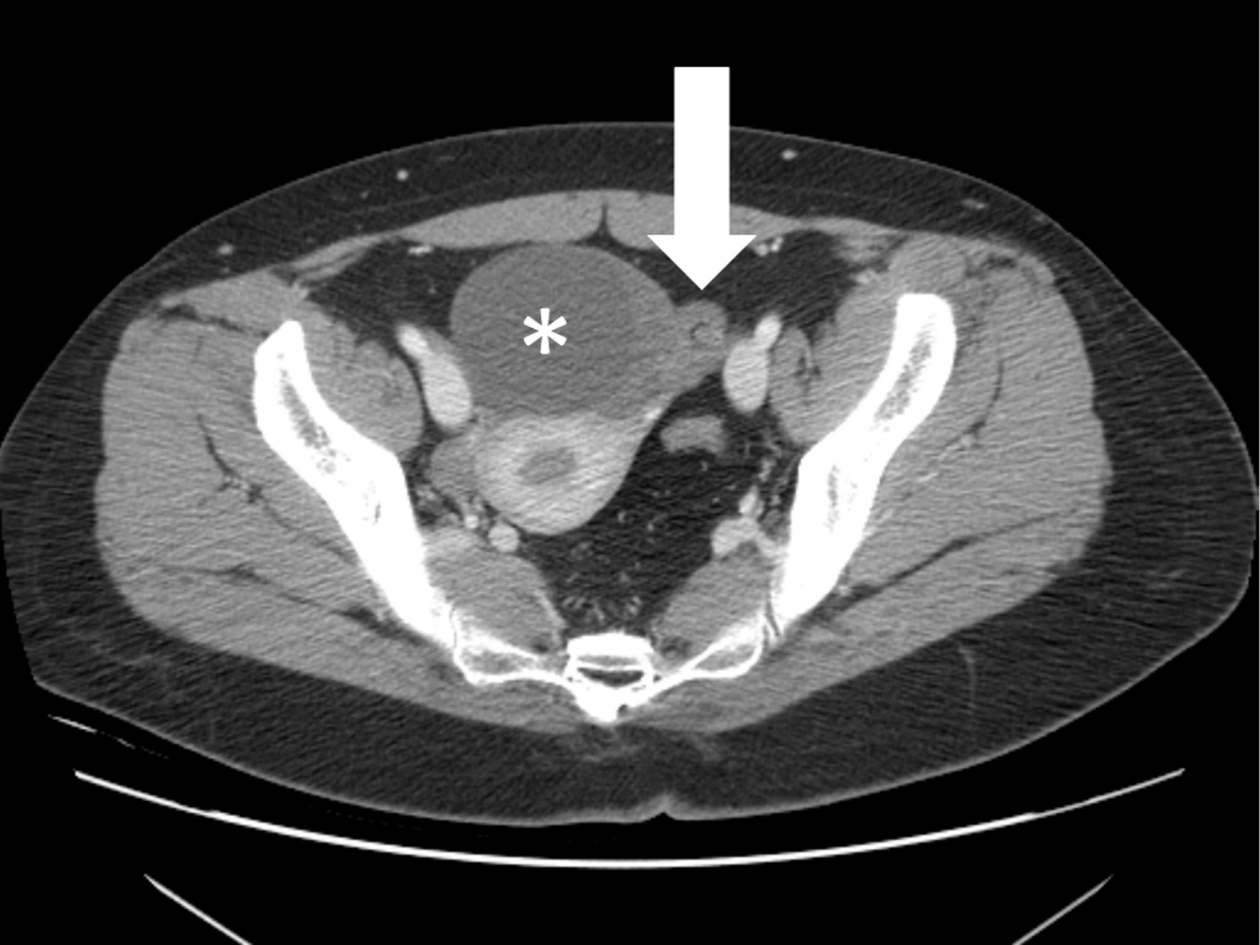
A 29-year-old woman with torsed left ovarian mature cystic teratoma. Axial contrast-enhanced CT scan shows a thickened pedicle (arrow) between a left ovarian cystic mass (asterisk) and the left uterine cornu with helical swirling appearance, suggestive of a whirl sign.

**Fig 3:**
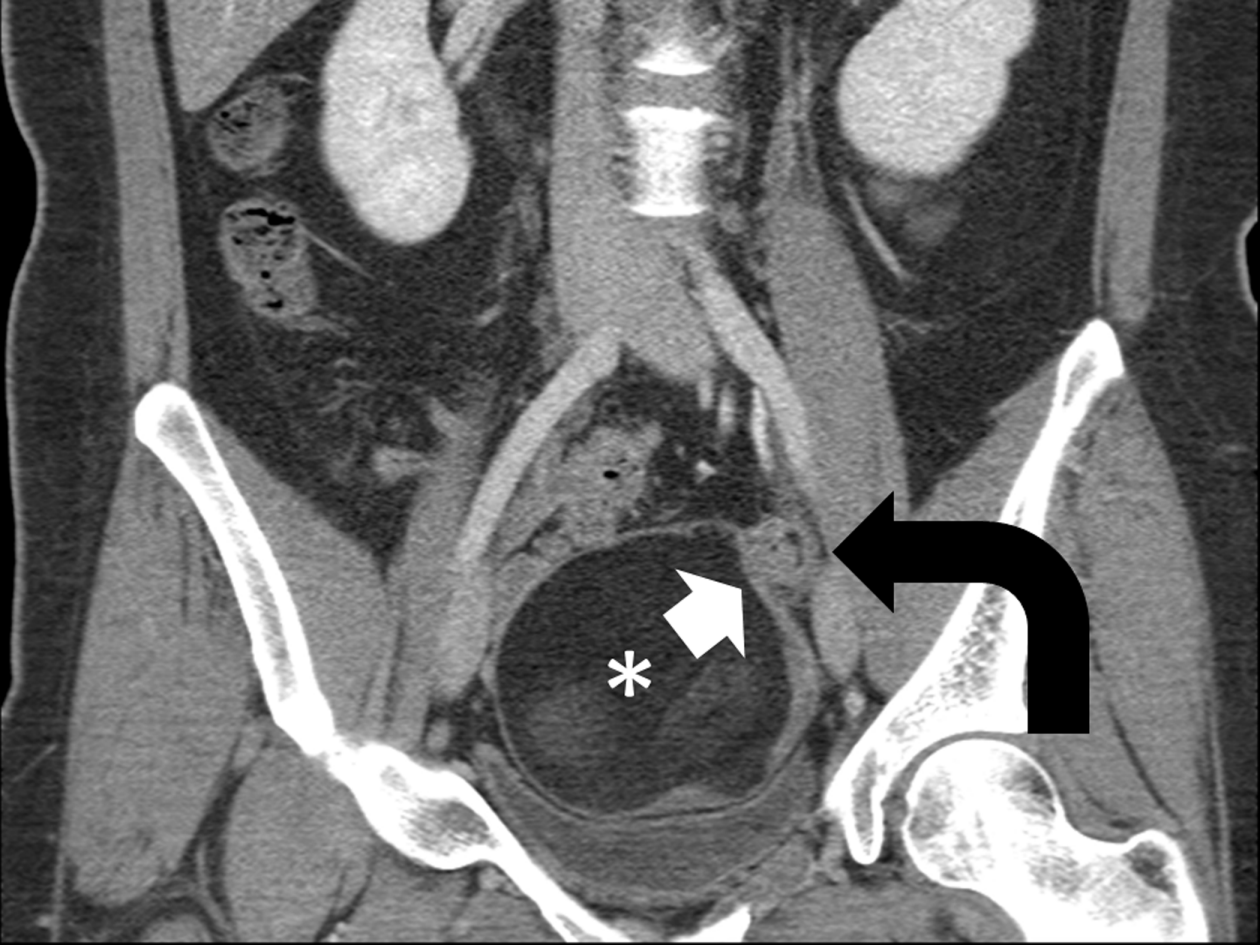
A 28-year-old woman with torsed left ovarian mature teratoma. Coronal contrast-enhanced CT scan shows a thickened pedicle (curved arrow) left superior to the fatty mass (asterisk). Denting (arrow) of the mass surface at the attachment site of the thickened pedicle is suggestive of a navel sign. Note that the thickened pedicle also shows a whirl sign.

**Fig 4:**
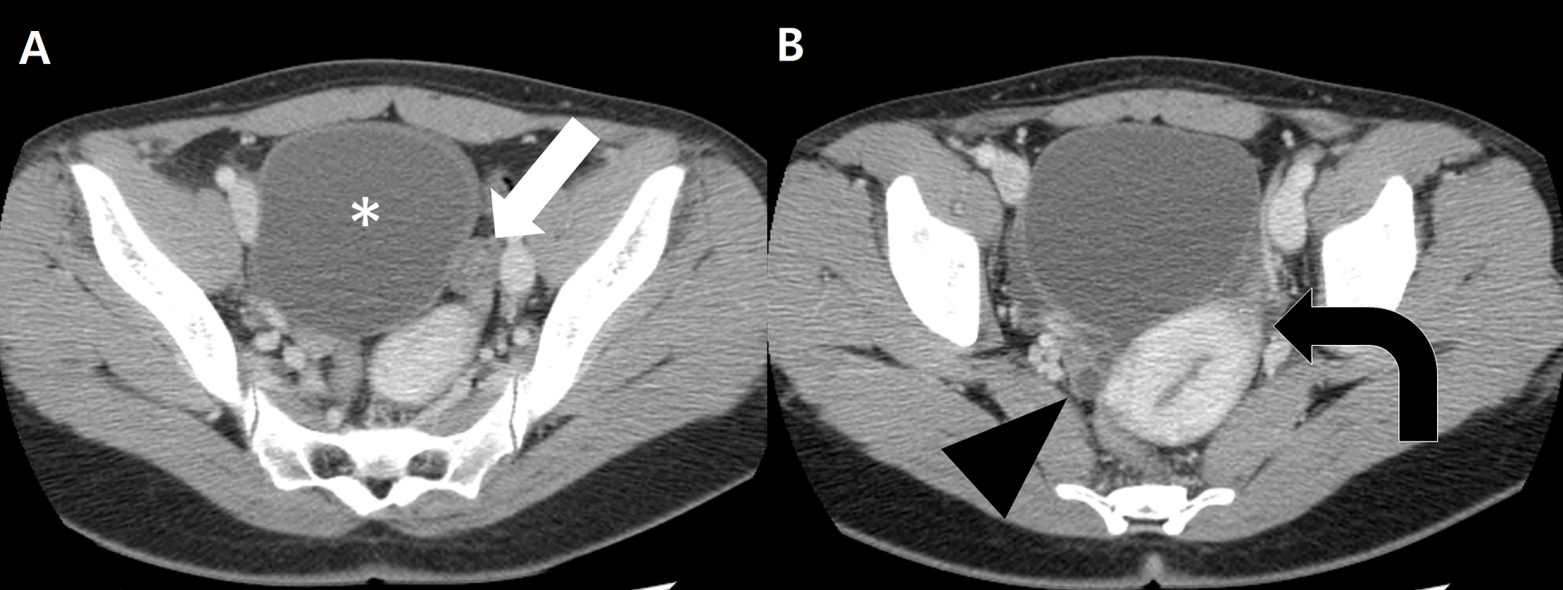
A 23-year-old woman with torsed left ovarian mucinous cystadenoma. A, Axial contrast-enhanced CT scan shows a thickened pedicle (arrow) between the left uterine cornu and a pelvic mass (asterisk). B, CT scan obtained caudal to image A shows uterine deviation (curved arrow) toward the thickened pedicle. Note that the right ovary (arrowhead) is seen separately.

All CT images were reviewed retrospectively and independently on a picture archiving and communication system workstation (Marosis M-view; infinitt, Seoul, Korea) by two radiologists (M.S.L. [reader 1] and M.H.M. [reader 2] with 3 years and 14 years of experience in genitourinary imaging, respectively), who were informed of the primary aim of the study but blind to the clinical and surgical data of the study population. Prior to the study, both readers underwent a training session to standardize their criteria for CT findings of adnexal torsion. Five CT examinations from another study period were used for the training session. Using a standardized questionnaire, the readers reported the presence of the following CT findings: a) prominent peripheral follicles, b) uterine deviation towards the ovarian mass, c) a thickened pedicle without an ancillary finding, and d) a thickened pedicle with an ancillary finding. A thickened pedicle was defined as an amorphous solid mass between the ovarian mass and the ipsilateral uterine cornu [8]. A whirl sign, a navel sign, and uterine deviation facing a thickened pedicle were categorized as ancillary findings that reinforce the association of thickened pedicles with adnexal torsion (Figs 2–4). The navel sign was defined as denting or flattening of the affected ovarian surface at the presumed attachment site of the thickened pedicles. The enhancement of the ovarian parenchyma of the affected ovary was also evaluated. The regions of interest for ovarian enhancement were drawn to encompass as much remnant ovarian parenchyma as possible, and care was given to avoid partial-volume artifacts. Each drawing was repeated twice, and measured values were averaged for later analysis. No ovarian enhancement was defined as an increase of less than 10 Hounsfield units between unenhanced and portal venous phase scans.

### Statistical analysis

Inter-reader agreement was evaluated by using the Cohen kappa statistic: A kappa score of 0.20 or less than 0.20 was considered slight agreement; 0.21–0.40, fair agreement; 0.41–0.60, moderate agreement; 0.61–0.80, substantial agreement and 0.81–1.00, almost perfect agreement [14]. CT findings of adnexal torsion were compared between women with adnexal torsion and women without adnexal torsion by using the Chi square test. Receiver operating characteristics (ROC) analysis was used to evaluate the diagnostic performance of the identified CT findings of adnexal torsion. The areas under the ROC curves (AUCs) were evaluated as the measure of a diagnostic test's discriminatory power and compared by using a Delong test [15]. All statistical analyses were performed using MedCalc version 16.8.4 software (MedCalc, Ostend, Belgium). A p value of less than 0.05 was considered to indicate a statistically significant difference.

## Results

Conservative treatment was performed in 11 (25.6%) of the 43 women with adnexal torsion: detorsion with cystectomy in 10, and detorsion in only 1. Definitive treatment was performed in 32 women (74.4%): salphingo-oophorectomy in 31 and oophorectomy in 1. Surgery revealed ovarian masses in 42 of the 43 women with adnexal torsion and in 43 of the 43 women without adnexal torsion. One woman was proven to have torsion of the normal ovary. Final pathologies for the ovarian masses are summarized in Table 1. A specific diagnosis could not be made in 18.6% (8/43) of the women with adnexal torsion due to massive hemorrhagic necrosis. Abdominal pain was significantly more common in women with adnexal torsion than women without adnexal torsion (97.7% vs. 23.3%, P < .01), and the intensity of the abdominal pain was also more severe in women with adnexal torsion than that in women without adnexal torsion (mean scale; 5.4 vs. 3.0, P = .01). However, there was no significant difference between the groups in the duration of the abdominal pain (mean day; 5.3 days vs. 1.8 days, P = .56) (Table 2).

**Table 1:** Histologic diagnosis of the case and control women.

Diagnosis	Case (n = 43)	Control (n = 43)	*P* Value
Non-neoplastic condition	8 (18.6)	8 (18.6)	1.00
Normal ovary	1	0	
Paratubal cyst	1	1	
Functional ovarian cyst	3	0	
Endometriotic cyst	3	6	
Tubo-ovarian abscess	0	1	
Benign neoplasm	26 (60.5)	28 (65.1)	.66
Serous cystadenoma	3	4	
Mucinous cystadenoma	8	12	
Fibrothecoma	0	1	
Mature teratoma	14	10	
Serous adenofibroma	0	1	
Collison tumor ^a^	1	0	
Borderline or malignant neoplasm	1 (2.3)	7 (16.3)	.03
Serous borderline tumor	0	1	
Serous cystadenocarcinoma	0	1	
Mucinous borderline tumor	1	4	
Endometrioid adenocarcinoma	0	1	
Cystic mass of unknown nature ^b^	8 (18.6)	0 (0.0)	< .01

Note- The numbers in parentheses are percentages.

^a^ The collison tumor was composed of mucinous cystadenoma and serous cystadenofibroma.

^b^ A specific diagnosis could not be made due to massive hemorrhagic necrosis.

**Table 2:** Clinical features of the case and control women.

Clinical manifestations	Case (n = 43)	Control (n = 43)	*P* value
Abdominal pain	42 (97.7%)	10 (23.3%)	P < .01
Pain duration	5.3±18.5 days	1.8 ±2.4 days	P = .56
Pain intensity ^a^	5.4±2.5	3.0±1.4	P = .01

Note-Data for pain duration and pain intensity are mean day ± standard deviation and mean scale ± standard deviation.

^a^ Pain intensity was quantified by a using numerical rating scale (NRS). The NRS was available in 34 of 42 women with abdominal pain in the case group and 8 of 10 women with abdominal pain in the control group.

Chi square analysis revealed that the following CT findings were significantly more common in women with adnexal torsion than in women without adnexal torsion: uterine deviation toward the side of the affected ovary (*P* = < .01 for reader 1 and *P* = .02 for reader 2) and thickened pedicles with ancillary findings (*P* < .01 for both readers). The evaluation of the parenchymal enhancement of the affected ovary was possible in 31 (72.1%) and 31 (72.1%) of 43 women with adnexal torsion and in 19 (44.2%) and in 15 (34.9%) of 43 women without adnexal torsion for reader 1 and reader 2, respectively. The lack of ovarian enhancement was also more frequently seen in women with adnexal torsion than in women without adnexal torsion (83.9% vs. 15.8%, *P* < .01 for reader 1 and 87.1% vs. 3%, P < .01 for reader 2). Thickened pedicles without ancillary findings and prominent peripheral follicles showed no statistically significant differences between the two groups (Table 3).

**Table 3:** The distribution of CT findings between the case (n = 43) and control (n = 43) group.

CT findings	Reader 1			Reader 2		
	Case	Control	*P* value	Case	Control	*P* value
Prominent peripheral follicles	6 (14.0)	5 (11.6)	.75	5 (11.6)	3 (7.0)	.46
Uterine deviation	22 (51.2)	6 (14.0)	< .01	17 (39.5)	7 (16.3)	.02
Thickened pedicles without ancillary findings^a^	4 (9.3)	1 (2.3)	.17	7 (16.3)	6 (14.0)	.76
Thickened pedicles with ancillary findings^a^	32 (74.4)	1 (2.3)	< .01	31 (72.1)	1 (2.3)	< .01
Thickened pedicle with a whirl sign	21 (48.8)	0 (0.0)	< .01	15 (34.9)	0 (0.0)	< .01
Thickened pedicle with a navel sign	17 (39.5)	1 (2.3)	< .01	21 (48.8)	1 (2.3)	< .01
Thickened pedicle with uterine deviation^b^	22 (51.2)	0 (0.0)	< .01	17 (39.5)	0 (0.0)	< .01
No ovarian enhancement^c^	26 (83.9)	3 (15.8)	< .01	27 (87.1)	3 (20.0)	< .01

Note-Data are the number of women, and the numbers in parentheses are percentages

^a^ Ancillary findings include a whirl sign, a navel sign or uterine deviation.

^b^ Uterine deviation as an ancillary finding was defined as uterine deviation facing the thickened pedicle.

^c^ The evaluation of the parenchymal enhancement of the affected ovary was possible in 31 (72.1%) and 31 (72.1%) of 43 women with adnexal torsion and in 19 (44.2%) and in 15 (34.9%) of 43 women without adnexal torsion for reader 1 and reader 2, respectively. No ovarian enhancement was defined as a difference of less than 10 Hounsfield units between unenhanced and portal venous phase scans

Table 4 shows the diagnostic performance of the identified CT findings in the diagnosis of adnexal torsion. Comparison of the AUCs revealed that thickened pedicles with ancillary findings were significantly superior to other identified CT findings or their combinations in the diagnosis of adnexal torsion (uterine deviation, *P* < .01; uterine deviation OR thickened pedicles with ancillary findings, *P* < .01; uterine deviation AND thickened pedicles with ancillary findings, *P* < .01 for both readers). When thickened pedicles with ancillary findings were used as a diagnostic criterion for adnexal torsion, we could achieve a sensitivity, specificity, positive likelihood ratio, negative likelihood ratio and accuracy of 74.4%, 97.7%, 32.00, 0.26 and 86% for reader 1 and 72.1%, 97.7%, 31.00, 0.29 and 85% for reader 2 (Table 4). For reader 1, the addition of ovarian non-enhancement as a diagnostic criterion for adnexal torsion made additional diagnosis in 6 of 11 false-negative women possible but resulted in 3 false-positive women. For reader 2, the addition of ovarian non-enhancement made additional diagnosis in 7 of 12 false-negative women possible but resulted in 3 false-positive women. The histologic diagnosis for the false-positive women is mature cystic teratoma (n = 1), mucinous borderline tumor (n = 1) and endometriotic cyst (n = 1) in reader 1 and mature cystic teratoma (n = 2) and fibrothecoma (n = 1) in reader 2.

**Table 4:** Diagnostic performance of individual and combined CT findings of adnexal torsion.

CT findings	Sensitivity	Specificity	Positive LR ^a^	Negative LR ^a^	AUC
Uterine deviation					
Reader 1	51.16 (35.5–66.7)	86.05 (72.1–94.7)	3.67 (1.7–8.1)	0.57 (0.4–0.8)	0.69 (0.58–0.78)
Reader 2	39.53 (25.0–55.6)	83.72 (69.3–93.2)	2.43 (1.1–5.3)	0.72 (0.5–1.0)	0.62 (0.51–0.72)
Thickened pedicle with ancillary findings^b^					
Reader 1	74.42 (58.8–86.5)	97.67 (87.7–99.9)	32.00 (4.6–223.8)	0.26 (0.2–0.4)	0.86 (0.77–0.93)
Reader 2	72.09 (56.3–84.7)	97.67 (87.7–99.9)	31.00 (4.4–217.0)	0.29 (0.2–0.5)	0.85 (0.76–0.92)
Uterine deviation OR thickened pedicle with ancillary findings^b^					
Reader 1	74.42 (58.8–86.5)	83.72 (69.3–93.2)	4.57 (2.3–9.2)	0.31 (0.2–0.5)	0.79 (0.69–0.87)
Reader 2	72.09 (56.3–84.7)	81.40 (66.6–91.6)	3.87 (2.0–7.4)	0.34 (0.2–0.6)	0.77 (0.66–0.85)
Uterine deviation AND thickened pedicle with ancillary findings^b^					
Reader 1	51.16 (35.5–66.7)	100.00 (91.8–100.0)		0.49 (0.4–0.7)	0.76 (0.65–0.84)
Reader 2	39.53 (25.0–55.6)	100.00 (91.8–100.0)		0.60 (0.5–0.8)	0.70 (0.59–0.79)

Note: Numbers in parentheses are 95% confidence intervals

^a^ LR means likelihood ratio. Positive LR for uterine deviation and thickened pedicle with ancillary findings cannot be determined because there is no false positive.

^b^ Ancillary findings include a whirl sign, a navel sign or uterine deviation facing thickened pedicles.

Interobserver agreement for the presence of CT findings of adnexal torsion was substantial or almost perfect, with weighted kappa values of 0.823 (95% CI 0.629–1.000) for prominent peripheral follicles, 0.835 (95%CI 0.709–0.961) for uterine deviation, 0.699 (95% CI 0.051–0.848) for thickened pedicles, 0.791 (95% CI 0.633–0.949) for a whirl sign, and 0.805 (95% CI 0.657–0.954) for a navel sign, respectively.

## Discussion

Adnexal torsion is defined as a twisting of adnexal structures including the ovary, the fallopian tube and their supporting structures [16]. Torsion of adnexal structures generally involves both the ovary and the fallopian tube because the wide attachment of the mesovarium or mesosalphinx to the rest of the broad ligament acts as a fulcrum [16–18]. Twisting of adnexal structures leads to anatomical changes that can be depicted on imaging studies including twisted pedicles, uterine deviation towards the affected ovary, prominent peripheral follicles, and decreased ovarian enhancement [4–8, 17].

**Fig 5:**
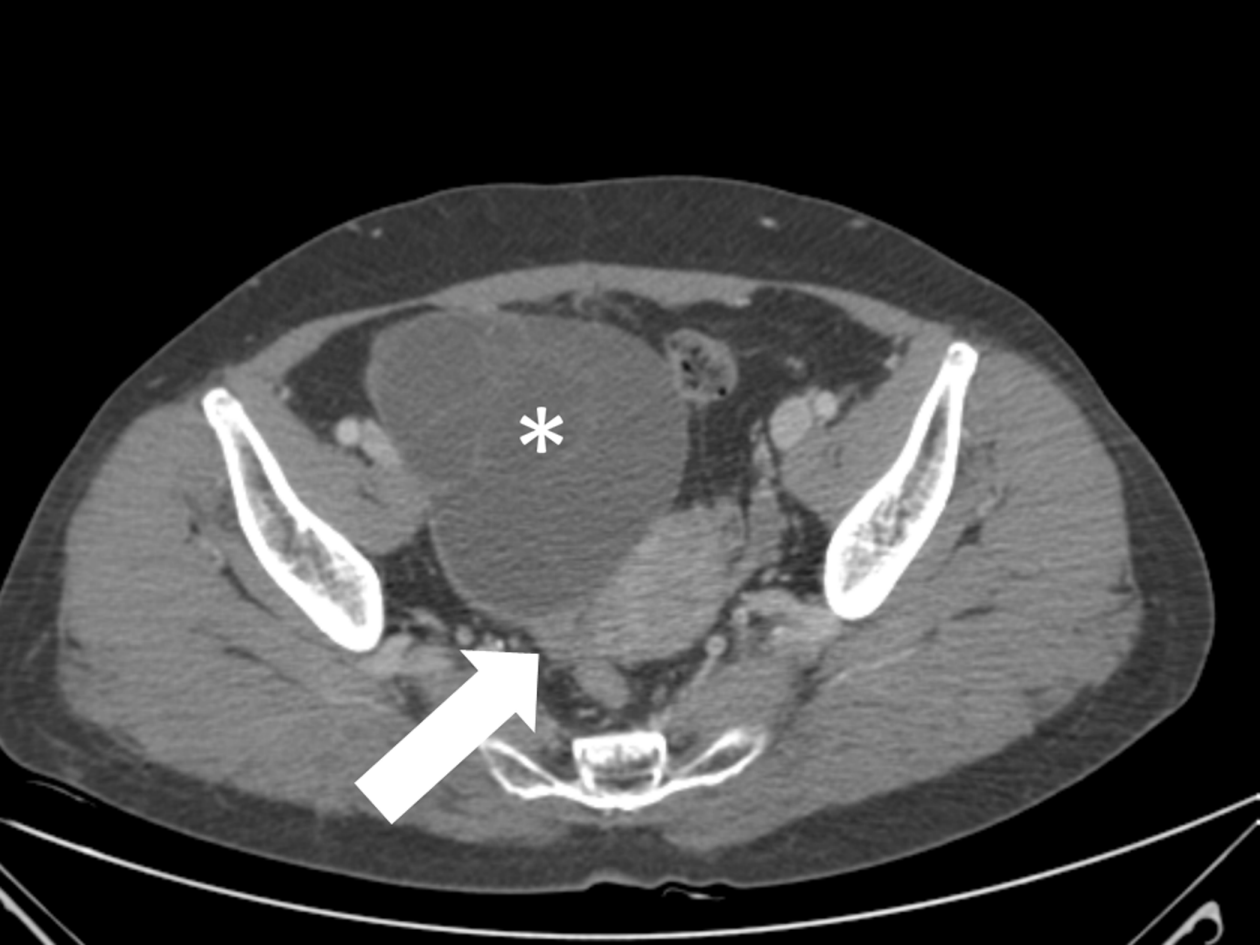
A 58-year-old woman with right ovarian mucinous cystadenoma. Axial contrast-enhanced CT scan shows a thickened pedicle (arrow) between a right ovarian cystic mass (asterisk) and the right uterine cornu. The thickened pedicle is not associated with a whirls sign or a navel sign. Uterine deviation is also not noted (not seen here). Surgery revealed a right ovarian cystic mass without torsion.

Twisted pedicles may consist of various components including the mesovarium, fallopian tube, broad ligaments, suspensory ligament, ovarian vessels and uterine vessels. Previous studies dealing with CT findings of adnexal torsion described a twisted pedicle as a tubal thickening or a whirl sign [5, 8]. In a study of 25 women with surgically proven adnexal torsion, Rha et al. [8] reported that tubal thickening in the form of an amorphous solid mass around the adnexal mass was noted in 75% (15/20) of the patients and a whirl sign with a target-like appearance in 10% (2/20) of the patients. In Hiller et al.’s study of 35 women with surgically proven adnexal torsion [5], they reported that tubal thickening was present in 17% (6/35) of the subjects and a whirl sign in 5.7% (2/35) of the subjects. Our understanding is that tubal thickening could be a thickened pedicle without a whirl sign or thickened fallopian tube itself. Because tubal thickening may also be seen in diseases other than adnexal torsion such as pelvic inflammatory disease [19, 20], we think tubal thickening itself cannot be a specific finding of adnexal torsion (Fig 5) and needs ancillary findings to be a specific finding of adnexal torsion (Figs 2–4).

**Fig 6:**
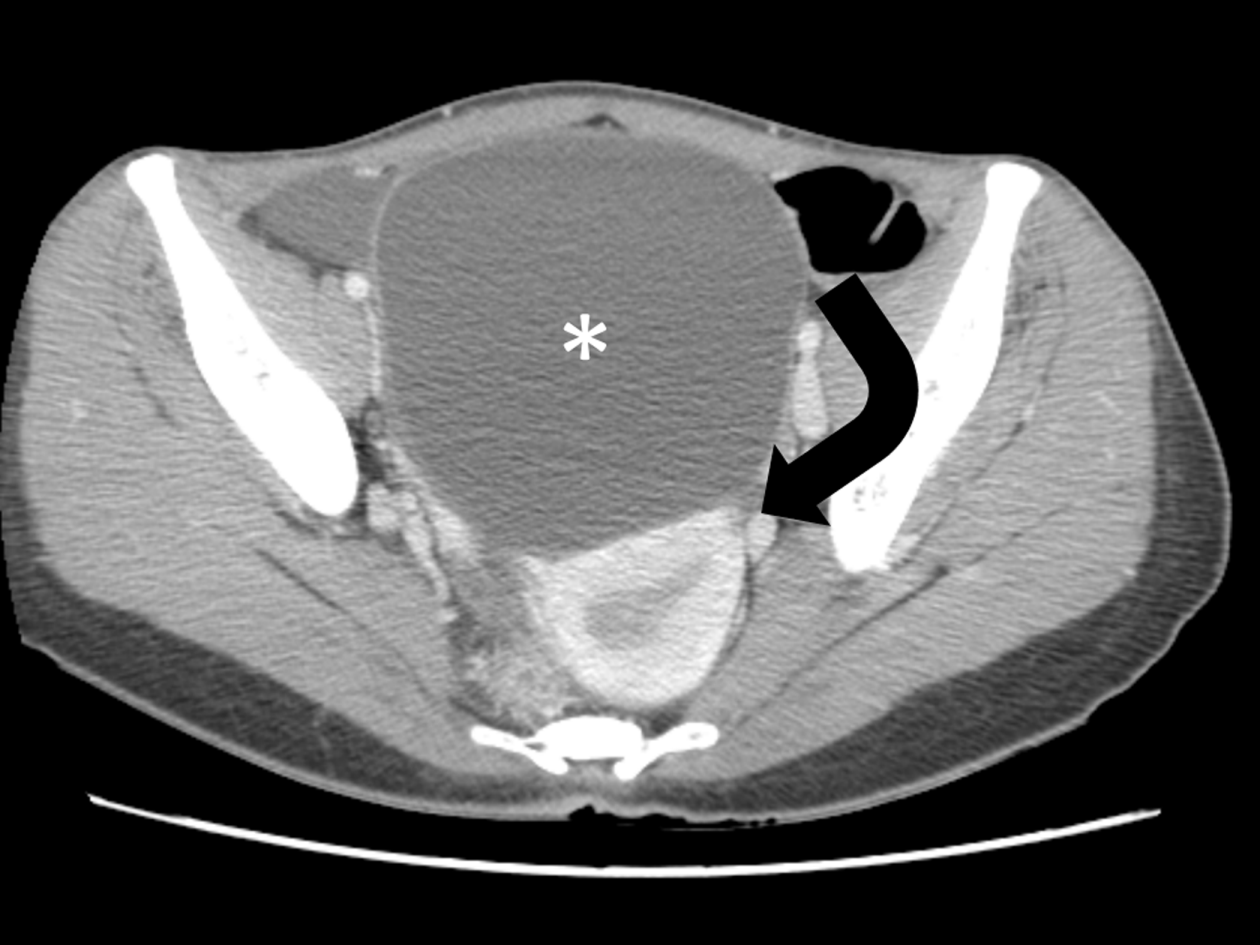
A 32-year-old woman with left ovarian mucinous cystadenoma. Uterine deviation (curved arrow) toward the pelvic mass (asterisk) is noted but there is no discernable thickened pedicle between the left uterine cornu and the pelvic mass. Surgery revealed a left ovarian cystic mass without torsion.

A whirl sign is a well-known ancillary finding and a thickened pedicle with a whirl sign has been recognized as a pathognomonic sign for adnexal torsion [4, 21]. In addition to the whirl sign, we added a navel sign and uterine deviation facing the thickened pedicle as ancillary findings. The navel sign is denting or flattening of the affected ovarian surface at the presumed insertion site of the thickened pedicles. Because twisting of the pedicles induces such denting or flattening on the ovarian surface, we thought that the navel sign could be a useful ancillary finding suggestive of pedicle twisting. This supposition proved true with our data that showed significant differences between women with and without adnexal torsion (39.5% vs. 2.3% for reader 1 and 48.8% vs. 2.3% for reader 2) (Table 3). Uterine deviation towards the affected ovary is a well-known CT finding of adnexal torsion. However, our experience has shown that ovarian masses may induce uterine deviation towards the affected ovary even in women without adnexal torsion (Fig 6). Uterine deviation needs additional findings to reduce false positive cases; thus, we adopted uterine deviation facing thickened pedicles as ancillary findings of thickened pedicles. In the present study, an ovarian mass could induce uterine deviation toward the affected ovary in the cases without adnexal torsion (6/43, 14.0% for reader 1 and 7/43, 16.3% for reader 2), and the application of uterine deviation facing thickened pedicles led to a significant reduction in the false positive rate (14.0% vs. 0.0% for reader 1 and 16.3% vs. 0.0% for reader 2) with little sensitivity loss (51.2% vs. 51.2% for reader 1 and 39.5% vs. 37.2% for reader 2) (Table 3).

A twisted pedicle is the pathognomonic sign of adnexal torsion but it is identified in less than one third of women with adnexal torsion on CT [4]. We extended such a pathognomonic sign into thickened pedicles with a navel sign and thickened pedicles with uterine deviation as well as thickened pedicles with a whirl sign. This effort has nearly doubled the sensitivity of CT in the diagnosis of adnexal torsion (from 48.8% to 74.4% for reader 1 and from 34.9% to 72.1% for reader 2), with little loss in the specificity (from 100% to 97.7% for both readers) (Table 3). Uterine deviation towards the affected ovary was also proven to be a CT finding of adnexal torsion. However, the combination of uterine deviation towards the affected ovary with thickened pedicles with ancillary findings did not increase the diagnostic value of thickened pedicles with ancillary findings in the diagnosis of adnexal torsion.

Lack of enhancement is also expected to be one of CT findings suggestive of adnexal torsion because ovarian torsion leads to obstruction to venous outflow and arterial inflow. Lack of ovarian enhancement sometimes can be difficult to distinguish from non-enhancing cysts and an enlarged ovary with a mass often has no measurable solid portion for evaluation of enhancement. However, to our knowledge, it is not known about how much ovarian enhancement can be assessed quantitatively on CT scans in women with suspicion of adnexal torsion. In the present study, the evaluation of ovarian enhancement was possible in 72.1% of women with adnexal torsion and in 34.9% - 44.2% of women without adnexal torsion. When the evaluation of ovarian enhancement was possible, the lack of ovarian enhancement was more frequently seen in women with adnexal torsion than in women without adnexal torsion (83.9% vs. 15.8%, *P* < .01 for reader 1 and 87.1% vs.3%, P < .01 for reader 2). Although considerable missing values made it difficult to compare ovarian enhancement with other CT findings, we think that ovarian non-enhancement could be also helpful in the diagnosis of adnexal torsion when the evaluation of ovarian enhancement is possible.

Prominent peripheral follicles, one of the CT findings of ovarian torsion, were not common findings in our study. Furthermore, there was no significant difference in the presence of prominent peripheral follicles between women with adnexal torsion and women without adnexal torsion (14.0% vs. 11.6%, *P* = .75 for reader 1 and 11.6% vs. 7.0%, *P* = .46 for reader 2). Prominent peripheral follicles are thought to result from peripheral displacement of follicles due to ovarian stromal edema [17]. Lee et al [18] found in a retrospective review of 116 women with surgically proven adnexal torsion that women without a detectable ovarian mass showed ovarian edema more frequently than women with a visible ovarian mass. They suggested smaller ovarian stroma reduced by an ovarian mass as the reason for low detectability of ovarian edema. The same is presumed to be true for the low prevalence of prominent peripheral follicles in the present study. The majority of our study population had ovarian masses; thus, a smaller stromal portion may be affected by outflow obstruction. This could be responsible for the low prevalence of prominent peripheral follicles in our study population. The relatively low tissue contrast of CT compared to MR or US may be another explanation for such a result.

Our study has several limitations. First, our study is limited by its retrospective nature, with the potential for selection and verifications biases. The choice of a consecutive sample of women with oophorectomy or salphingo-oophrectomy as control women is another limitation of our study because selection of an enriched sample of women with non-painful masses may have biased our results. A third limitation is that the interval between imaging and surgery was lengthy for some women and the ovary may torse or detorse over time. We think this is an unavoidable limitation given that the episodes of pain can occur for several days to months before admission in some women with adnexal torsion [16]. A fourth limitation is that coronal reformation of acquired images was not available in some women. Because the use of coronal reformation may improve the detection of adnexal torsion [22], this limitation could influence the delineation of the CT findings we analyzed. Although readers were blind to the clinical and surgical data of the study population, they were informed of the primary aim of the study, and the level of interrogation of CT findings of adnexal torsion may have been higher than it would be when reading a CT in a routine clinical situation. This is another limitation of our study.

In conclusion, a thickened pedicle with ancillary findings is the most accurate CT predictor of adnexal torsion. In addition to the previously recognized whirl sign as a strong ancillary predictor of torsion, we found the addition of a navel sign, and uterine deviation facing the thickened pedicles, when seen with thickened pedicles, can increase the sensitivity of CT in the diagnosis of adnexal torsion. We hope that our results will be helpful in the management of women with adnexal torsion in whom early recognition is important to preserve the affected ovary and prevent serious complications.

## Supporting information

S1 File Original data in.xlsx spreadsheets. (XLSX)
